# 水产品中微塑料的污染现状及检测方法研究进展

**DOI:** 10.3724/SP.J.1123.2025.02013

**Published:** 2025-08-08

**Authors:** Tingting LOU, Lin HUANG, Youzhi SU, Jun LIU, Haitao LI, Pinyao ZHAO

**Affiliations:** 1.天津科技大学生物工程学院，天津 300457; 1. College of Bioengineering，Tianjin University of Science and Technology，Tianjin 300457，China; 2.伊宁海关技术中心，新疆 伊宁 835000; 2. Technical Center of Yining Customs，Yining 835000，China; 3.成都海关技术中心，四川 成都 610041; 3. Chengdu Customs Technology Center，Chengdu 610041，China; 4.天津市理化分析中心有限公司，天津 300051; 4. Tianjin Physical and Chemical Analysis Center Co. Ltd. ，Tianjin 300051; 5.宜宾学院，四川 宜宾 644000; 5. Yibin University，Yibin 644000，China

**Keywords:** 微塑料, 水产品, 污染现状, 检测方法, microplastics, aquatic products, pollution status, detection methods

## Abstract

微塑料作为新兴环境污染物，已成为当前国际社会重点关注的“四大类新污染物”之一。人类活动密集、渔业资源丰富的海岸带地区已成为微塑料污染的重灾区。微塑料颗粒通过食物链进入水生生物体内，在贝类、甲壳类及鱼类等水产品中显著富集。由于水产品是人类获取动物蛋白的重要来源，微塑料污染有可能借助食物链传递，对人类健康造成不可逆转的损害。本文系统综述了水产品中微塑料污染的研究现状，深入分析其主要来源、污染形态、分布特征及对人类健康的潜在威胁。同时，重点探讨了国内外水产品中微塑料提取方法与鉴别检测技术方面的研究进展，客观评价了各类方法的优缺点及适用范围。此外，文章对水产品中微塑料鉴别检测技术的未来发展趋势进行了前瞻性展望。通过全面梳理和总结现有研究成果，本文旨在为水产品中微塑料的分析鉴定技术提供科学参考，为制定微塑料污染防控策略、推动检测技术创新以及促进水产养殖业可持续发展，提供理论支撑和实践指导。同时，为实现全球水产品的安全监管、加强环境保护提供科学基础，并为水产品食品安全风险评估和微塑料污染的监测治理，提供有力的理论依据和技术支持。

## Abstract (en)

As an emerging pollutant， microplastics have become a significant component of global environmental pollution， thereby attracting growing attention from the scientific community and policymakers. Microplastics， defined as plastic particles smaller than 5 mm in diameter， are widely distributed in aquatic environments， including rivers， lakes， oceans， and even groundwater. Due to their small size and persistent nature， microplastics can be easily ingested by aquatic organisms， particularly shellfish， crustaceans， and fish， which are integral components of the aquatic food web. These aquatic products serve as a crucial source of animal protein for human consumption， making the contamination of microplastics in these organisms a direct pathway for human exposure. Once ingested， microplastics may accumulate in human tissues and organs， potentially causing irreversible harm to human health， including inflammatory responses， oxidative stress， and even carcinogenic effects. Therefore， understanding the contamination， distribution， and ecological impacts of microplastics in aquatic products is of paramount importance for both environmental protection and public health. This paper provides a comprehensive review of the current state of microplastic pollution in aquatic products， focusing on the primary sources， pollution pathways， distribution patterns， and ecological consequences. Microplastics enter aquatic environments through various routes， including the breakdown of larger plastic debris， industrial effluents， wastewater treatment plants， and agricultural runoff. Once in the water， microplastics can be ingested by aquatic organisms， either directly or indirectly through the consumption of contaminated prey. The accumulation of microplastics in aquatic organisms not only affects their growth， reproduction， and survival but also disrupts the balance of aquatic ecosystems. Furthermore， microplastics can act as carriers for other pollutants， such as heavy metals and persistent organic pollutants， exacerbating their toxic effects on aquatic life and humans. In addition to analyzing the sources and ecological impacts of microplastic pollution， this paper critically evaluates the technical progress in microplastic extraction and identification methods. Current techniques for microplastic detection include visual identification， spectroscopic methods（e.g.， Fourier-transform infrared spectroscopy（FT-IR） and Raman spectroscopy， and chromatographic-mass spectrometric approaches（e.g.， pyrolysis-gas chromatography-mass spectrometry（Py-GC-MS））. Each method has its advantages and limitations. For instance， visual identification is simple and cost-effective but lacks accuracy for small-sized particles， while spectroscopic methods offer high specificity and sensitivity but require expensive equipment and specialized expertise. Chromatographic-mass spectrometric techniques provide detailed chemical composition analysis but are often time-consuming and complex. This paper discusses the strengths and weaknesses of these methods， highlighting the need for standardized protocols to improve the comparability and reliability of microplastic detection data. Looking ahead， this paper prospects the future directions of microplastic identification and detection technologies in aquatic products. Emerging techniques， such as nanomaterial-based sensors， surface-enhanced Raman scattering（SERS）， and machine learning-assisted image analysis， hold great promise for enhancing the sensitivity， accuracy， and efficiency of microplastic detection. Moreover， the integration of multiple detection methods， such as combining spectroscopic and chromatographic techniques， could provide a more comprehensive understanding of microplastic contamination. The development of low-cost， portable detection devices is also essential for enabling widespread monitoring and real-time assessment of microplastic pollution in aquatic environments. Finally， this paper addresses the challenges and potential prevention and control measures in microplastic research. Key challenges include the lack of standardized detection methods， difficulties in detecting small microplastics or nanoplastics， and limited knowledge of their long-term ecological and health impacts. To tackle these issues， interdisciplinary collaboration among scientists， policymakers， and industry stakeholders is crucial. Potential prevention and control measures include reducing plastic waste at the source， improving wastewater treatment technologies， and promoting public awareness of plastic pollution. By summarizing existing research findings， this paper aims to provide a theoretical foundation and technical support for the risk assessment of microplastic contamination in aquatic products and the development of effective monitoring and management strategies. The insights gained from this review will contribute to safeguarding aquatic ecosystems， ensuring the safety of aquatic products， and protecting human health from the adverse effects of microplastic pollution.

随着全球塑料生产和消费的持续增长，塑料污染已成为全球环境保护和生态健康的重大挑战。塑料污染物，尤其是具有持久性环境赋存特征的微塑料，已成为全球环境科学领域关注的焦点^［1-3］^，并被列为国际公认的“四大类新污染物”之一^［4，5］^。微塑料通常指尺寸小于5 mm的塑料颗粒，主要包括塑料产品在生产、使用消费和废弃过程中形成的小颗粒以及大型塑料物体经物理、光或生物降解后产生的碎片颗粒^［6］^。微塑料因其持久性、低密度和广泛分布的特性，能够在水环境中长时间悬浮，并通过水体流动或生物摄取进入浮游生物和鱼类、贝类等水产品体内。水产品是人类获取蛋白质和微量营养素的重要来源，其安全性直接关系到全球食品安全和人类健康。微塑料污染不仅会影响水生生物的生长、繁殖和健康，还可能通过食物链传递，最终影响人类食品安全，进而对人类健康构成潜在威胁^［7-9］^。因此，水产品中微塑料污染的检测与控制，已成为全球环保和食品安全监管的关键议题。

研究表明，微塑料在海洋特别是海岸带区域、河流入海口、湖泊等水环境介质中呈现普遍性分布^［10］^。海岸带是陆地和海洋的过渡区域，作为第一海洋经济区，既是人类活动最为频繁的区域，也是鱼类、贝类等水产品的主要栖息地和养殖区。值得注意的是，水产养殖过程中大量使用的塑料设施（如渔网、浮标、输水管等），在紫外辐照和机械磨损作用下会持续释放微塑料，成为重要的次生污染源。国内外研究显示，鱼类和双壳类等经济水产品中普遍存在微塑料污染，其赋存浓度与聚合物类型呈现显著的空间异质性^［11］^。微塑料颗粒通过摄食进入水生生物体内，并可能在消化系统中积累，甚至进入其肌肉组织^［12］^。其生物毒性效应与颗粒粒径、形态、表面特性及化学添加剂浸出行为密切相关，可能引发水生生物消化系统损伤、炎症反应和氧化应激等病理现象，如消化系统阻塞、营养吸收受阻和组织损伤等^［13］^，已成为生态毒理学研究的重要方向。

近年来，随着全球微塑料污染问题的加剧，相关研究呈现跨学科快速发展的态势。基于Web of Science核心合集数据库的文献计量分析（检索策略：主题词=microplastic*，时间跨度=2015-2025）显示，该领域近10年间累计发表近2万篇研究论文，从学科分布来看，环境领域发文量占比最高（68.3%），其次为生态和毒理学（21.3%），表明微塑料的环境归趋与生态效应是当前研究重点。而在此研究过程中必不可少的表征定量分析方法的作用更加凸显。其分析方法学的发展呈现显著的技术迭代特征。从研究初期以目视法、光学显微镜等传统光学法发展到傅里叶变换红外光谱（μ-FT-IR）和拉曼光谱法等单一光谱技术再到以光谱法、色谱-质谱法、高光谱成像等联用技术；当前阶段则开始整合人工智能图像识别算法，通过AI与智能学习，使检测更加精准和科学。

尽管近年来针对水产品中微塑料污染的研究已取得显著进展，但该领域仍面临诸多挑战。首先，在分析检测层面，现有检测技术方法尚不完善。现有的微塑料分析方法面对水产品复杂基质中微塑料的检测仍存在灵敏度不足的问题。其次，方法学标准化程度不足导致数据可比性受限。不同研究团队采用的微塑料分离提取和检测方法各异，可能会对检测结果的准确性造成影响，这直接制约着污染源解析和环境归趋研究的可靠性。本文系统梳理了近年来发表的相关研究，综述了水产品中微塑料污染现状及其检测方法的最新进展。通过综合分析现有研究的成果和不足，期待为未来微塑料污染的防控策略制定、检测技术创新以及水产养殖业的可持续发展提供理论支持和实践指导，为实现全球水产品的安全监管和环境保护提供科学依据。

## 1 水产品中微塑料的污染现状

### 1.1 水产品中微塑料污染的来源

水产品中微塑料污染的主要来源包括陆地塑料废弃物的流入、渔网、浮标等渔业工具的使用、养殖设施中的塑料材料以及水产养殖过程中塑料污染物的排放等。这些塑料制品在水生生态系统中可通过微生物（如蜡样芽孢杆菌、微球菌属或棒状杆菌）、热、氧化、光或水解的作用缓慢降解，或经过机械和风化过程等物理作用转化为微塑料。饲料中的微塑料颗粒也是水产品中微塑料污染的重要来源^［14］^。此外，水产品在加工过程中，也会因引入塑料制品而造成水产品的微塑料污染^［15］^。

### 1.2 水产品中微塑料的形式与分布

根据文献报道，微塑料在水产品中的形式多样，主要表现为纤维状、碎片状、薄膜、颗粒状及球状^［16-18］^。这一现象主要与水生生物所处环境中微塑料的存在形式密切相关。水产品中检测到的微塑料形态主要受到水生生物（如鱼类、贝类）摄入行为和生长过程的影响。水生生物通过进食和呼吸随机摄入塑料，因此它们体内微塑料的形态分布与地表水和沉积物中的微塑料相似。多项研究表明，地表水和沉积物中微塑料的主要形状是纤维状、碎片状和薄膜^［19-21］^。在海洋环境中，纤维状和颗粒状微塑料较为常见。而在水产养殖环境中，纤维状微塑料更为突出，其主要来源于渔网及其他养殖设施的塑料材料。

微塑料在水产品中的分布因生物种类而异，主要受水生动物生理结构、摄食方式及微塑料环境行为的影响。鱼类体内的微塑料主要积累在胃肠道和鳃部，与其摄食方式和呼吸机制密切相关。鱼类通过主动摄食摄入微塑料，常误将其当作食物或随食物摄入。研究表明，鱼类对颜色鲜艳（如蓝色、黑色）或形状类似浮游生物的微塑料具有选择性摄食倾向^［22］^。Rochman等^［23］^研究发现，鱼类消化道中的微塑料主要来源于误食，消化系统无法分解微塑料，导致其在胃肠道中长期滞留并富集。鱼类的鳃是呼吸和滤水的重要器官，微塑料（尤其是纤维状颗粒）易随水流被鳃丝截留。贝类体内的微塑料主要分布在外壳和滤食器官（如鳃和消化腺），与其滤食习性和生理结构密切相关。贝类外壳表面粗糙，易通过静电作用或物理吸附截留微塑料，尤其是在高浓度或湍流水体环境中。作为滤食性生物，贝类通过鳃和消化腺过滤水体中的悬浮颗粒物，易误食粒径较小的微塑料。贝类消化系统无法分解微塑料，导致其在滤食器官中积累。

此外，微塑料的粒径、形状和密度会影响其在水体中的分布及被生物摄取的效率。例如，纤维状微塑料更容易被鱼类鳃部截留，而小粒径微塑料更容易被贝类滤食器官摄取。

### 1.3 水产品中微塑料的种类与颜色

研究表明^［16，24，25］^，鱼类等水产品中最常见的5种聚合物类型为聚乙烯（PE）、聚酰胺（PA）、聚丙烯（PP）、聚对苯二甲酸乙酯（PET）、聚苯乙烯（PS）。这种现象是由于PE、PP和PS等材料被广泛应用于捕鱼工具以及食品包装材料，如塑料薄膜、塑料袋和瓶装容器等^［24，26，27］^。PET则广泛应用于包装、纺织和薄膜等领域。PA通常用于渔业活动中，如渔网和其他渔业工具的制造。这些塑料材料通过意外损失、污水排放以及捕鱼和人类活动等途径在海洋环境中累积^［26］^。

鱼类、贝类等水生生物体内的微塑料最常见颜色为蓝色和黑色，其次是透明、白色、红色和绿色^［16，24，28］^。这可能与这些颜色的塑料在工业生产和个人消费品中的广泛使用有关。例如，蓝色塑料常见于渔网、绳索、包装材料以及合成纤维衣物中，而黑色塑料则多用于汽车轮胎、工业涂料以及电子设备外壳等。这些材料在使用过程中容易破碎成为微塑料进入水体，成为水生生物摄入的主要来源。而透明和白色微塑料常见于广泛使用的塑料袋、食品包装、一次性餐具等。而红色和绿色塑料可能来源于特定的工业产品、纺织品或塑料制品，但其使用量相对较少，因此在生物体内的检出率也较低。微塑料的环境行为与生物选择性也影响着不同颜色微塑料的检出率，例如不同颜色的微塑料可能具有不同的密度和浮力特性，进而影响其在水体中的分布和行为。此外，当前微塑料检测方法（如目视鉴定法），可能存在对某些颜色微塑料的识别偏差，这也可能影响研究结果中不同颜色微塑料的检出率。因此，开发更精准的微塑料检测方法至关重要。

### 1.4 水产品中微塑料的含量与分布

水产品中微塑料污染的程度因地区而异。微塑料在水产品中的含量在不同水域和水产养殖模式下存在较大差异，这主要与其所处水生环境中的微塑料含量相关。此外，微塑料污染还呈现出季节性变化。在渤海湾的研究中，春季和秋季水产品中的微塑料丰度较高，而冬季和夏季的污染程度较低，这一现象与气候变化、海流速度及水温等因素的季节性波动密切相关^［29］^。

国内学者Li等^［30］^调查了中国12 400英里海岸线沿线22个地点的贻贝（*Mytilus edulis*）微塑料污染情况，贻贝样品中总微塑料含量从0.9～4.6个/g不等。野生贻贝中微塑料含量（2.7个/g）高于养殖贻贝的微塑料含量（1.6 个/g）。人类活动密集地区的贻贝微塑料含量为3.3 个/g，显著高于人类活动较少地区（1.6 个/g）。Ding等^［17，18，31］^研究了从青岛、厦门、东营等地采集的贝类和鱼类的微塑料污染情况，结果显示：市售养殖海产品超过80%的个体和40%的野生个体中检出微塑料。不同市场购买的扇贝（*Chlamys farreri*）中微塑料平均含量为3.2~7.1 个/g（消化系统湿重），贻贝（*Mytilus galloprovincialis*）微塑料含为2.0~12.8 个/g，且养殖贻贝的微塑料含量高于野生贻贝。此外，对比青岛和厦门商品贝类消化系统中微塑料的污染水平，发现青岛不同采样点的贝类样品中70%~100%检测到微塑料，厦门贝类不同采样点的样品中70%~90%检测到微塑料，其中青岛贝类微塑料含量为0.8～4.4 个/g，厦门贝类微塑料含量为2.1～4.0 个/g。风险评估显示，青岛商业贝类消化系统中的微塑料污染对人类健康构成的潜在风险高于厦门。采自青岛和东营市场的双壳类和鱼类的软组织和消化道检测显示，鱼类微塑料个体检出率高于双壳类，且以单个物品为单位测量的微塑料含量在鱼类中显著更高。Jia等^［32］^调查了中国沿海17个地区的牡蛎发现，84%的采样牡蛎含有微塑料，平均含量为0.62个/g，表明不同沿海地区养殖牡蛎普遍存在微塑料污染，但其含量与其他国家相当或较低。此外，调查结果还显示南部沿海地区微塑料含量高于华北地区的区域特征。

世界其他各地都有不同水产品中的微塑料污染情况报道。Su等^［33］^报道了澳大利亚墨尔本地区鱼类的微塑料污染情况，调查的鱼类中有19.4%的样品检出了微塑料，每个鱼样品中微塑料含量为0.6个，且头部含量普遍高于其他部位；Abidli等^［34］^对突尼斯北部湖泊6种软体动物的微塑料污染水平调查表明，湖中软体动物普遍存在微塑料污染，体内微塑料总含量为（0.70±0.11）～（1.48±0.02） 个/g。Danopoulos等^［12］^通过系统分析27个地区软体动物的微塑料含量，发现其范围为0～10.5 个/g，甲壳类动物为0.1～8.6 个/g，鱼类为0～2.9 个/g。Hermabessiere等^［35］^研究了英吉利海峡海岸线（法国）的蓝贻贝（*Mytilus edulis*）和普通鸟蛤（*Cerastoderma edule*）的微塑料污染情况。结果显示，微塑料在这些水产品中的污染比例为34%~58%，含量为（0.15±0.06 ）~（0.74±0.35） 个/g。有学者^［36］^研究了来自北爱奥尼亚海（地中海）希腊水域的贻贝和鱼类等海产品的微塑料污染情况，发现在贻贝和沙丁鱼、赤鲷、羊鱼等3种鱼类中均发现了微塑料。贻贝中微塑料含量为1.7～2个/只，检出率为46.25%，而鱼类样品中微塑料含量为1.5～1.9个/只，其中沙丁鱼的微塑料检出率最高（47.2%）。Cho等^［37］^调查了韩国3个主要城市渔业市场的牡蛎、贻贝、蛤蜊、扇贝等双壳类动物的微塑料污染情况。发现样品中微塑料的平均含量为（0.15±0.20） 个/g，并估算韩国人口通过食用贝类摄入的微塑料年摄入量约为212 个/（人·年）。

以上研究表明，世界各地的水产品均受到不同程度的微塑料污染，对不同区域水产品微塑料污染情况进行调查分析有助于识别潜在的风险区域或物种，开展食品安全风险评估，并为后续研究和政策制定提供指导；同时也说明了持续监测和深入研究不同种类水产品微塑料污染的重要性。

### 1.5 水产品中微塑料对人类健康的潜在风险

水产品作为全球重要的动物蛋白来源，水产品中的微塑料污染不可避免地会影响食品安全，进而威胁到人类健康。微塑料颗粒通过食物链富集或其他途径进入人体后，可通过多途径引发人体多系统毒性，可能增强炎症反应，某些微塑料还可以在活细胞（如M细胞或树突状细胞）中转移至淋巴和/或循环系统，并在人体器官或组织内积累，随着时间的推移，可能对免疫系统、内分泌系统，甚至肝脏和肾脏等重要器官产生毒性影响^［38］^，主要表现有致癌作用、免疫毒性、肠道疾病、呼吸系统损伤、心血管毒性、生殖与发育毒性等^［39］^。另外，因微塑料具有疏水性，且化学结构中带有不同官能团，容易吸附多环芳烃、双酚A（BPA）等有机污染物和铅、砷、镉、汞等重金属有害物质^［40］^，这些有害化学物质可能通过微塑料颗粒进入人体，并对免疫系统、内分泌系统等造成长期影响^［41］^，进而导致多种癌症的发生^［39］^。

微塑料毒理学的研究表明^［42-45］^，其毒性机制（见图1）主要包括：（1）物理性损伤：微塑料可能引起物理应激和损伤，例如在肠道或组织中的摩擦或堵塞。（2）炎症和氧化应激：微塑料的存在可能导致炎症反应和氧化应激，损害细胞功能。（3）化学毒性：微塑料可能吸附环境中的有害化学物质（如多环芳烃、邻苯二甲酸酯、多氯联苯和铅），并在体内释放这些化学物质，导致毒性效应。（4）免疫反应：微塑料可能触发免疫系统的异常反应，导致免疫相关疾病。然而，目前关于微塑料对人体细胞的影响研究有限，且现有研究中使用研究模型与实际人体组织中的积累量还有一定的差异^［45］^。

**图1:**
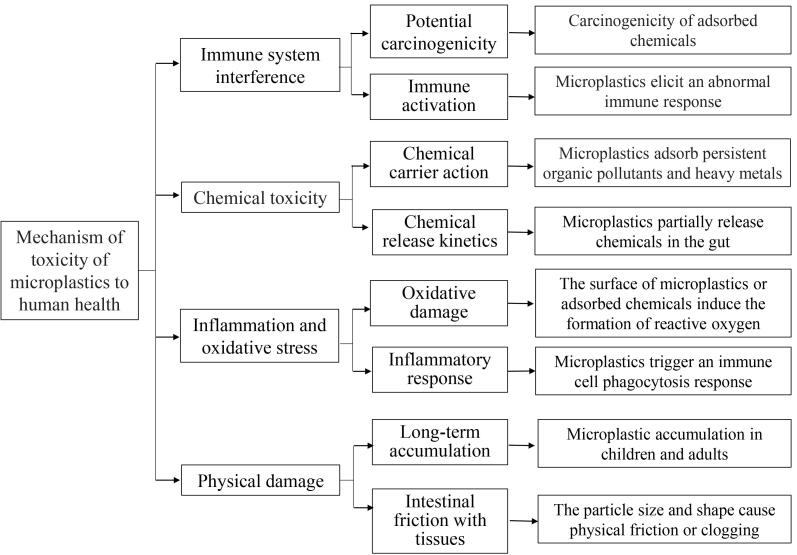
微塑料对人类健康的毒性机理示意图

微塑料对人类健康的影响受其粒径、化学组成及暴露剂量等因素共同作用。当前专家学者持续致力于积累更多的暴露数据、挖掘影响机制，并优化风险评估方法，提高微塑料暴露对人类健康风险的精准评估能力。其中，针对水产品中微塑料污染水平开展系统监测和科学风险评估，确保其符合食品安全标准，是保障人类健康的关键环节。

## 2 水产品中微塑料检测的前处理方法

对水产品中微塑料污染情况进行调查分析，需经历样品采集、样品处理、微塑料分离、鉴别分析等流程。与水样、土壤等环境样品不同，在对水产品等水生生物进行微塑料分析前，需对生物样品中的有机物进行消解处理，以提取出其中的微塑料用于后续鉴定。该处理过程既要求有效地消解有机物、去除干扰物质，又不能改变微塑料的重量、计数或形状。目前，水产品等生物样品中的有机物主要采用酸、碱、氧化剂或酶等化学试剂来消解。

### 2.1 酸消解法

酸消解法是利用酸性溶液来破坏有机物、溶解样品杂质，以释放纯净微塑料颗粒的常用样品前处理方法。消解液通常使用硝酸溶液。Thushari等^［46］^将双壳类/腹足类软体动物及甲壳类动物样品浸泡在69%浓硝酸溶液中，并煮沸2 h进行消解。Siddique等^［47］^使用65% HNO_3_溶液在70 ℃下对来自孟加拉湾圣马丁岛的热带鱼样品进行消解，通过密度分离提取微塑料，并评估了微塑料对热带鱼的潜在影响和危害。Wang等^［48］^使用65% HNO_3_溶液对南黄海海洋生物样品进行消解，检测到其生物组织中微塑料含量为1.7~47.0 个/g。Santana等^［49］^采用69% HNO_3_溶液，在室温条件下对贻贝组织进行过夜消化，随后加热煮沸15 min，实现了样品的有效消解。Claessens等^［50］^通过与盐酸、过氧化氢、氢氧化钠等消解法对比发现，硝酸消解法对待测生物样品的破坏率为94%~98%，消解效率最高。

除硝酸溶液外，高氯酸溶液也有应用，通常与硝酸溶液按一定比例混合使用。De Witte等^［51］^在分析贻贝样品时，对比了65% HNO_3_溶液和HNO_3_/HClO_4_混合溶液的消解效果，发现使用混合溶液消解组织中塑料纤维的检测效果更佳，单独使用HNO_3_溶液消解时，不仅无法完全消解有机物，还可能导致部分微塑料发生变色或解体，进而影响测定结果^［52］^。此外，有学者研究使用盐酸溶液^［53］^进行消解，但其效率较低，虽然加热可提高效率，但可能导致稳定性差的微塑料降解，影响测试结果。

### 2.2 碱消解法

碱消解法是利用强碱溶液使生物体中的有机物发生消解的方法，最常用的试剂为NaOH溶液和KOH溶液，其中使用最多的碱性溶液是10% KOH溶液。消解温度和消解时间显著影响反应速率。温度升高加速消解，在低温下（如30 ℃）延长消解时间显著提高消解程度；而在高温下，因短时间内即可基本完成消解，延长时间效果有限。Daniel等^［54］^使用了10% KOH溶液在60 ℃恒温环境中孵育24 h对印度沿海水域的白虾进行了有效的消解，并检测到样本虾体中微塑料平均含量为（0.39±0.6） 个/只，季风季节（7-8月）的微塑料污染水平显著高于其他月份。Karami等^［55］^在40 ℃条件下，利用10% KOH溶液对鱼肉和鱼片进行消化，结果显示消解效果良好，同时塑料聚合物保持完整。邹亚丹等^［56］^对6种消解方法进行了比较，发现10% KOH溶液消解效果最好，回收率高达96%。Akhbarizadeh等^［15］^利用10% KOH溶液在40 ℃恒温下对罐头鱼类（金枪鱼和鲭鱼）样品进行消解，调查了样品中微塑料的存在情况，并评估了其成分、潜在来源及人体摄入风险。Dehaut等^［57］^使用10% KOH溶液在60 ℃下消解贻贝、螃蟹和鱼组织，结果显示该消解液对生物组织进行了有效消化，除乙酸纤维素外，其他待测微塑料聚合物均无显著降解。Cole等^［11］^研究表明，NaOH溶液的消解效率已经达到90%。相较于酸消解法，碱消解法更加温和，对稳定性差的微塑料结构影响相对小，在应用上也最为广泛。

### 2.3 氧化剂消解法

氧化剂消解法通常是采用过氧化物（如H_2_O_2_）溶液对样品进行消解处理，其消解能力依赖于其产生的羟基自由基，因其氧化能力强，环境友好等特点，逐渐成为生物样品处理的重要选择。但是在低温下使用H_2_O_2_溶液样品难以消解完全，因此实验中一般采用高温进行消解。

一些研究者^［30-32，58-61］^采用30% H_2_O_2_溶液在65 ℃恒温振荡培养箱对贝类、鱼类和蟹类等样品进行消解，通过高温振荡快速分解样品的蛋白质、脂质等有机物，通过室温静置一段时间彻底清除残余有机碎片，均获得了良好的处理效果。然而，Claessens等^［50］^和Zhong等^［61］^通过实验比较了不同消解方式对贻贝组织中微塑料的降解时，发现PA6和PA66两种微塑料颗粒在双氧水消解法中的回收率为70%~95%左右，稍微受到影响，这可能加热消解过程中产生了泡沫导致部分物质损失。但是相较酸、碱消解法，过氧化物消解法在微塑料完整性保护，复杂基质适应性和操作安全性方面具有显著优势，尤其适用于高脂鱼类、软体动物等样品的处理。

### 2.4 酶消解法

使用生物酶对生物组织进行消解的研究相对较少，且所使用的酶类不尽相同。Courtene-Jones等^［62］^测试了多种蛋白水解酶（胰蛋白酶、木瓜蛋白酶和胶原酶），旨在确定对生物样品的最佳消化效果并评估各类酶对微塑料的影响；结果显示，使用胰蛋白酶可快速有效地从生物样品中提取微塑料，且不会改变微塑料的理化性质。Catarino等^［63］^比较了NaOH溶液、35% HNO_3_和蛋白酶等3种消解法提取贻贝软组织中微塑料的效率，结果表明三者均可实现对样品组织的完全消解，但HNO_3_处理会对微塑料造成一定程度的破坏。添加至贻贝的微塑料在NaOH和酶消解处理后的回收率相近。von Friesen等^［64］^报道了一种基于胰腺酶与pH缓冲液的酶解方法，该方法可有效消解生物组织，且其效率高于氢氧化钾消解法。根据Cole等^［11］^的研究，酶消解法可降解约97%的有机物。与化学消解法不同，该方法不会导致聚合物的溶解或降解，且酶本身无危险性。其缺点是处理时间较长，条件要求比较苛刻（需满足每种酶的最佳温度和pH条件）且成本相对较高。

水产品样品的消解前处理不仅要考虑化学试剂的选择，还需关注消解时间、温度、分离步骤以及与其他化学试剂和过滤器孔径的匹配。除上述酸、碱、氧化剂、酶消解法外，还有使用混合溶剂（如KOH+H₂O₂、NaClO+CH_3_OH等）的消解方式。其中，10% KOH溶液消解法应用最为广泛，这可能与其条件温和，对微塑料结构影响小有关。

此外，随着对微塑料研究的深入，静电分离、淘析柱优化和磁性萃取等新型处理方法正逐步应用于微塑料的提取和分离^［5］^，但目前仍缺乏检测生物样品或环境样品中微塑料的标准化方法，这是微塑料污染检测与分析面临的挑战。由于不同研究采用的采样和提取方法差异较大，数据的可比性和一致性受到严重影响，进而限制了对微塑料污染全球分布、来源及环境影响的全面了解。

## 3 水产品中微塑料的鉴定及检测方法

微塑料种类多样，化学组成复杂，粒径分布广泛，导致其精准鉴定与分析面临诸多挑战。目前，主流检测技术主要包括化学组分分析、聚合物结构表征和物理形态鉴定3类，不同方法在灵敏度、分辨率及适用场景方面存在显著差异。已报道的方法中，色谱-质谱联用技术主要用于微塑料成分的定性定量分析；红外光谱法和拉曼光谱法常用于官能团结构分析；而光学显微镜与扫描电子显微镜主要用于微塑料表面形貌的观察。

### 3.1 色谱-质谱技术

由于色谱-质谱分析可获取微塑料的准确结构和相对分子质量，因此在微塑料鉴定分析中得到研究者的广泛认可，具有广阔的应用前景。应用该技术时，需先将提取后的微塑料在高温下分解为小分子物质，再结合热分析技术进入色谱柱检测。目前较常用的色谱-质谱法有热解气相色谱-质谱（pyrolysis-gas chromatography-mass spectrometry，Py-GC-MS）、热萃取解吸-气相色谱-质谱（thermo-extraction and desorption coupled with gas chromatography/mass spectrometry，TED-GC-MS）及液相色谱-串联质谱（liquid chromatography-mass spectrometry/mass spectrometry， LC-MS/MS）等。

Py-GC-MS的原理是在惰性气体环境中加热微塑料，使其裂解为聚合物片段，并依据片段大小和极性差异在色谱柱中分离，随后通过质谱检测器进行分析^［65］^，依据热解产物的质谱特征与标准图谱数据库进行比对，可鉴定微塑料聚合物的类型^［8，66，67］^。该方法对微塑料粒径无严格要求，但属于破坏性检测，无法提供其物理形态等表征信息。因此，该方法主要用于微塑料的化学成分鉴定及聚合物降解产物的识别，同时能够将微塑料成分与其他化学物质分离。Peters等^［68］^利用Py-GC-MS技术鉴定了墨西哥湾沿岸海鱼胃内容物中的微塑料颗粒，在检测到的43种微塑料中，聚氯乙烯（PVC）占比最高（32.6%），其次为PET（9.3%）、PA（9.3%）、硅胶（2.3%）和环氧树脂（2.3%）。Ribeiro等^［69］^使用Py-GC-MS对澳大利亚高商业价值的水产品牡蛎、虾、鱿鱼、螃蟹和沙丁鱼可食用部分的PS、PE、聚氯乙烯、PP和聚甲基丙烯酸甲酯等微塑料成分进行鉴定和定量分析，发现所有样品均检测出聚氯乙烯，PE的含量为0.04～2.4 mg/g，其中沙丁鱼的总微塑料含量最高（0.3 mg/g组织），鱿鱼最低（0.04 mg/g组织）。Sefiloglu等^［70］^利用Py-GC-MS技术对尼罗罗非鱼（*Oreochromis niloticus*）肌肉组织中的PE、PP、PS 和 聚甲基丙烯酸甲酯（PMMA）4种微塑料进行定性与定量分析，在42%的样品中检测到微塑料，平均含量为（0.14±0.32） μg/g。Zhong等^［71］^通过碱消化法去除水生贝类中的有机物，以六氟异丙醇为萃取溶剂，结合红外光谱、激光粒度测定和扫描电子显微镜分析碱处理前后的样品，再利用Py-GC-MS对微塑料进行分析；经色谱分离和质谱鉴定后，对高温裂解产生的特征离子碎片进行定量分析，该方法对PA6和PA66的线性范围为2～64 μg/g，定量限分别为0.6和2.0 μg/g。

TED-GC-MS结合了热重分析（TGA）和GC-MS分析技术，利用TGA装置对微塑料聚合物进行热解，产生的气态分解产物经固相萃取吸附后，再解吸附进入GC-MS进行鉴定分析^［72］^。与Py-GC-MS相比，TED-GC-MS的一大优势在于可直接对复杂基质样品进行分析，通常无需繁琐的前处理步骤^［73］^。目前，TED-GC-MS已广泛应用于水体、土壤等相对简单的环境样品分析^［74-76］^，在水产品中微塑料的检测与鉴定方面也具有较大的发展潜力。Liu等^［77］^利用TED-GC-MS鉴定和量化了海洋贻贝中的微塑料，发现中国沿海6个地点的紫贻贝组织中微塑料含量高达1.71 mg/kg（平均值为0.58 mg/kg），其中PE最为丰富。此外，该方法还研究了塑料相关添加剂的存在及其与微塑料的相互作用，发现含有PMMA的微塑料可吸附环境污染物苯并（*k*）荧蒽（BkF），从而减少鱼类对污染物的吸收。Kittner等^［78］^开发了一种基于聚合物特异性热分解产物作为标记化合物的TED-GC-MS快速检测方法，对8种微塑料成分聚合物进行加标检测，并检测出了两种可生物降解聚合物聚己二酸对苯二甲酸丁二醇酯（PBAT）和聚丙交酯（PLA）。

LC-MS/MS与气相色谱-质谱类似，通常在进样前需对样品进行提取、纯化及解聚，随后通过色谱分离与质谱分析，实现对样品中微塑料相对分子质量的推断。然而，由于复杂生物样品在热解过程中可能产生不完全降解物质，污染电离源并造成仪器损坏，该方法尚未在生物基质中得到广泛应用。目前，仅有少数研究采用该方法对食品样品中的微塑料进行检测。例如，Di Giacinto等^［79］^从地中海捕获的剑鱼（*Xiphias gladius*）和蓝鳍金枪鱼（*Thunnus thynnus*）可食用肌肉中提取微塑料颗粒，包括PET、聚碳酸酯（PC）、BPA和对邻苯二甲酸（PTA）。提取的微塑料颗粒经立体显微镜与拉曼显微光谱表征后，进一步采用LC-MS/MS对聚合物（如PET和PC）进行鉴定。

尽管质谱分析技术无法直接提供微塑料的物理形状及数量信息，且其分析过程具有破坏性，常导致样品无法进行后续形貌分析。然而，质谱分析技术结合热分析方法在微塑料检测中展现出独特优势：所需样品量少（通常仅需0.1~5 mg），对于基质较为单一的样品，可实现“进样-热解-检测”一体化自动流程，可显著提高检测效率，并有效区分塑料添加剂等干扰成分。此外，该类方法在推动微塑料检测的标准化和数据一致性提升方面具有显著的应用潜力。未来，可通过建立统一的塑料裂解产物特征数据库，开发智能化谱图解析算法，提升对改性塑料和降解产物的识别能力，并结合高分辨质谱与机器学习技术，进一步增强该方法的选择性和检测通量，为深入解析微塑料的环境行为机制提供坚实的技术支撑。

### 3.2 光谱检测技术

传统的微塑料分析技术主要依赖光谱法，包括红外光谱法和拉曼光谱法等。这些方法通过测量特定特征官能团的吸收带来实现对微塑料的鉴别。

#### 3.2.1 傅里叶变换红外光谱法（FT-IR）

FT-IR通过测量微塑料颗粒的红外吸收光谱来推断其化学结构，具有前处理简单、无损分析等优势。该方法可依据谱图信息对微塑料进行鉴别，是目前最常用的光谱分析技术之一，也是微塑料检测中不可或缺的重要工具。Danopoulos等^［12］^通过检索MEDLINE、EMBASE 和Web of Science 数据库（分别从1947年、1974年和1900年建库起）至2020年10月的所有报道海洋食品中微塑料含量的研究，发现FT-IR被广泛用来鉴定和检测微塑料的含量。Dinka等^［36］^采用FT-IR分析了地中海希腊水域水产品的微塑料摄入情况，发现贻贝（*Mytilus galloprovincialis*）和3种鱼类（*Sardina pilchardus*、*Pagellus erythrinus、Mullus barbatus*）体内均检测到微塑料。Pantoja等^［80］^利用FT-IR调查分析了亚马逊河口养殖牡蛎的微塑料污染情况，发现PA纤维颗粒占大多数（91%），其次是PS碎片（9%），且肝胰腺和性腺比其他部位积累更多微塑料。Abidli等^［34］^借助FT-TR技术，针对从比塞大泻湖采集的6种商业软体动物开展了微塑料污染调查工作，发现微塑料含量为（703.95±109.80）~（1 482.82±19.20） 粒/kg，检测出纤维、碎片和薄膜等3种有色微塑料，尺寸范围为0.1~1 mm，并通过FT-IR分析证实了PE和PP两种聚合物的存在。此外，光学光热红外（O-PTIR）显微光谱作为一种新兴技术，已被用于表征微塑料表面亚微米尺度的化学变化^［81］^。其原理是用可见激光和脉冲中间红外激光同轴照射样品表面，红外吸收区温度升高导致折射率发生变化，进而改变可见激光在该区域的传播路径，从而获得样品的红外光谱。利用O-PTIR对样品表面微观区域进行扫描，可绘制红外吸收光谱及图像，从而实现对纳米塑料粒径分布及数量的直观识别与统计分析^［82］^。

#### 3.2.2 拉曼光谱法

拉曼光谱法亦是当前应用广泛的微塑料检测技术之一，可通过测量微塑料颗粒的拉曼散射信号来识别其化学成分^［83］^。与FT-IR相似，该方法具有无损、样品量少、高通量及环境友好等优点，可测量含有荧光且对光不稳定的化合物，且扫描速度快、谱图重现性好，能在较小粒度下实现高精度分析并能有效区分塑料类型。Hermabessiere等^［35］^利用拉曼光谱技术调查英吉利海峡海岸线（法国）蓝贻贝和普通鸟蛤中的微塑料污染情况，发现双壳类微塑料污染比例为34%~58%，蓝贻贝和普通鸟蛤中微塑料含量为（0.15±0.06）~（0.74±0.35） 个/g。Thushari等^［46］^利用拉曼光谱分析了从泰国东海岸收集的贝类中的微塑料。Pitt等^［84］^综合应用FT-IR与拉曼光谱对大西洋旗鱼体内的微塑料进行分析，鉴定出PA、PE、PP和聚氨酯等聚合物颗粒。然而，拉曼光谱检测小尺寸微塑料时，因固有散射截面低，灵敏度受到一定的限制。近年来，基于表面等离子体共振理论的表面增强拉曼（SERS）光谱技术发展迅速。该方法可通过金银纳米颗粒或SERS衬底来增强纳米塑料的拉曼信号，甚至可通过SERS探针标记纳米塑料模型颗粒，来追踪其在环境和生物体中的分布^［85］^。该技术已成功应用于对PS、PET、PMMA等微塑料成分的检测，检测粒径低至100 nm，有望在水产品中小尺度微塑料分析中展现更大潜力。随着技术不断发展，拉曼光谱法可与多种技术联用（如空间外差技术、荧光成像技术、差分拉曼光谱技术和共焦显微拉曼技术）以实现更加精准的检测，例如Dong等^［86］^建立的微塑料快速检测联用技术已成功用于实际样品分析。然而，拉曼光谱法对样品的制备要求较高，易受到荧光干扰。激光激发可能引发样品局部加热，导致部分微塑料聚合物降解，从而限制了其在大规模样品检测中的广泛应用。

### 3.3 显微成像技术

显微镜法主要通过观察样品中微塑料颗粒的形态特征，实现对其定性或定量分析。常见方法包括普通光学显微镜法、荧光显微镜法和扫描电子显微镜（SEM）法。

光学显微镜法是最常用的显微镜技术之一，适用于较大颗粒的微塑料检测，可有效观察微塑料颗粒形态并进行初步定性分析^［59，87，88］^。Mohsen等^［89］^采用荧光显微镜对水产品饲料中的微塑料含量进行了检测，并对其形状、颜色、尺寸和聚合物类型进行了系统表征。然而，该方法对小于1 mm的微塑料颗粒的检测效果较差，通常需与其他技术联用以提高检测效果。

SEM在微塑料分析中具有重要应用，尤其在微塑料颗粒的表面特征分析中发挥着关键作用，结合能量色散光谱技术（EDS）可同步获取微塑料的元素组成，为溯源研究提供多维数据支撑。Akhbarizadeh等^［15］^不仅采用荧光显微镜对罐头鱼（吞拿鱼与鲭鱼）中的微塑料进行定量分析，还结合拉曼显微镜与SEM-EDS识别其聚合物类型与组成；结果表明，80%的样品中至少检测到一个微塑料颗粒，其中以纤维为主要形态，PET（32.8%）为最常见的聚合物成分。Wang等^［48］^利用SEM成功识别了水产品中微塑料颗粒的表面形态（如粗糙表面、破损边缘和明显孔隙）。Ding等^［18］^结合连续立体显微镜、配备衰减全反射（μ-ATR-FT-IR）的微傅里叶变换红外光谱与SEM-EDS，建立了高精度微塑料检测系统。显微成像技术可提供微塑料颗粒的高分辨率图像，可对微塑料的数据进行验证，但其缺点是设备成本高，通常需依赖人工判读，存在主观误差且难以实现高通量分析，主要适用于实验室场景。

综上所述，不同微塑料鉴别分析方法各有侧重：部分方法可在不破坏污染物原貌的前提下进行检测，但准确性有限；部分方法仅适用于定性分析；另有方法虽能精确测定成分及含量，但检测过程具破坏性，限制了样品的后续分析。因此，实际工作中可利用两种甚至多种联用技术（如在线热重分析-傅里叶变换红外反射-质谱法（TGA-FT-IR-MS）^［90］^）来实现样品中微塑料的分离鉴别及定量检测。TGA可以对微塑料进行定量测定并可以分析其分解行为和动力学，FT-IR和色谱-质谱法可表征热解产物，从而更全面分析微塑料的裂解行为和结构特征。随着检测技术的发展，越来越多的新型联用技术被应用于微塑料检测。然而，目前微塑料的鉴别与检测仍缺乏统一标准化方法，建立科学、可行的检测标准已成为亟需解决的问题，这对于保障检测数据的可比性与可靠性、推动全球微塑料污染监测和治理具有重要意义。表1列举了部分文献中报道的水产品的前处理方法和微塑料的鉴别检测方法。表2总结了不同方法的优缺点。

**表1:** 部分已报道的水产品的前处理方法和鉴别检测方法汇总

No.	Seafood types	Pretreat solvents/methods	Qualitative and quantitative methods	Abundance of MPs	Ref.
1	coral reef fish	69% HNO_3_	FT-IR	4.38-10 particles/g	［^47^］
2	rock oyster	69% HNO_3_	RS	0.2-0.6 counts/g	［^46^］
3	shellfish	10% KOH	FT-IR	0.8-4.4 items/g（Qingdao）； 2.1-4.0 items/g（Xiamen）	［^31^］
4	*Chlamys farreri* and *Mytilus* g*alloprovincialis*	10% KOH	micro FT-IR， stereo microscope	*Chlamys farreri*： 3.2-7.1 items/g； *Mytilus galloprovincialis*： 2.0-12.8 items/g	［^17^］
5	mussels， crabs and fish	10% KOH，60 ℃，24 h	microscope， py-GC-MS， and RS	/	［^57^］
6	crabs，fish， oysters	KOH-NaI	FT-IR	0-11 particles/individual	［^91^］
7	mussels and three fish species	30% H_2_O_2_	FT-IR	1.7-2 items/individual in mussels， 1.5-1.9 items/individual in fish	［^36^］
8	blue mussel and common cockle	10% KOH	RS	（0.15±0.06）-（0.74±0.35） MP/g	［^35^］
9	bivalve species， oyster， mussel， manila clam and scallop	10% KOH	RS	mean（0.15±0.20） n/g and（0.97±0.74） n/individual	［^37^］
10	*Chlamys farreri* and *Mytilus galloprovincialis*	10% KOH	FT-IR	3.2-7.1 MP/g and 2.0-12.8 MP/g	［^17^］
11	*Fundulus heteroclitus*	10% KOH	FT-IR， RS	（85.5±70.2） and（11±12.5） MP/g	［^84^］
12	commercial molluscs	10% KOH	FT-IR	（703.95±109.80）-（1482.82±19.20） items/kg	［^34^］
13	canned fish	10% KOH	microscope， SEM-EDX	at least 1 items/sample（80% samples）	［^15^］
14	eastern oysters； Atlantic mud crabs	30% H_2_O_2_	microscope	4.2 pieces in tissues/individual； 16.5 microplastic pieces/individual	［^59^］
15	fish	10% KOH	RS	/	［^92^］
16	fish	10% KOH	Py-GC-MS	/	［^68^］
17	fish	KOH and H_2_O_2_	FT-IR	/	［^93^］
18	fish	NaClO，CH_3_OH	microscope	/	［^94^］
19	fish	NaClO，CH_3_OH	microscope	/	［^88^］
20	*Mytilus edulis*	enzymatic	SEM， FT-IR	（1.05±0.66）-（4.44±3.03） MP/g	［^62^］

FT-IR：Fourier transform infrared spectroscopy； RS： Raman spectroscopy； py-GC-MS：pyrolysis-gas chromatography-mass spectrometry； SEM-EDX： scanning electron microscope-energy dispersive X-ray spectroscopy. n/g and MP/g： number per gram and microparticles per gram； /： not mentioned.

**表2:** 微塑料不同检测方法的比较

Detection method	Advantages	Limitations	Main applications
Microscopy	low cost， simple operation， suitable for morphological analysis	low sensitivity to small particles	preliminary screening， morphology analysis
FT-IR	can identify chemical components， widely used in plastic identification	complex samples may interfere， limited sensitivity	chemical composition analysis， plastic identification
Raman spectroscopy	high sensitivity， suitable for detecting small particles	high cost， complex operation	analysis of nanoparticles and small particles
Chromatography-mass spectrometry	can identify types of microplastics， suitable for larger particles	cannot provide morphological information of particles	identification of plastic types， quantitative analysis

## 4 结语

随着全球塑料污染问题日益严峻，微塑料污染已成为水产品安全与生态环境保护领域亟待应对的关键议题。微塑料不仅对水生生物构成威胁，还可能通过食物链富集进入人体，对人类健康产生潜在风险。近年来，微塑料检测技术已从传统的光谱方法发展到色谱-质谱分析方法，显著提升了微塑料的鉴别与检测能力。然而，现有方法在标准化程度、检测灵敏度、成本控制及小尺寸微塑料颗粒检测等方面仍面临挑战^［95］^。

为应对这些挑战，未来研究应聚焦以下方向：（1）创新样品前处理方法：针对水产品等生物样品基质的复杂性，发展基于功能材料（如磁性纳米材料、分子印迹聚合物等）的吸附分离技术，结合固相萃取和超声波辅助萃取等先进样品前处理技术，提高微塑料的提取效率，有效降低基质干扰。（2）优化高灵敏度色谱-质谱技术：提升色谱分离能力，结合高分辨率质谱或多级质谱技术，实现对微塑料特征碎片的精准识别与定量分析，从而显著提升检出限和定量限，为痕量微塑料的检测提供可靠的技术支撑。（3）推动纳米技术与高效传感器结合：基于纳米传感器的检测平台结合光谱技术可显著提高对纳米级颗粒的灵敏度，开发高效传感器是提高小尺寸微塑料检测灵敏度和准确性的关键^［96］^。例如利用纳米颗粒结合SERS技术，可增强微塑料信号，实现微米级甚至纳米级颗粒的识别与定量^［95，97］^。（4）加强多种鉴别检测技术联用：单一方法难以满足微塑料复杂性和多样性的分析需求，未来应将物理、化学、光谱及生物检测技术结合应用。例如，显微成像、红外光谱与质谱分析联用，可全面评估微塑料种类、浓度及生态风险，为污染防控提供可靠数据^［90］^。（5）研发低成本检测设备：现有技术灵敏度高，但设备成本限制了其大规模应用。未来应开发低成本传感器技术，如集成光谱传感器和便携式红外光谱仪，降低检测成本并实现现场快速检测，支持广泛普及与实时监测。（6）构建标准化、智能化和自动化监测系统：建立标准化方法，通过集成高效传感器与AI算法，可建立实时数据采集与分析系统，实现微塑料污染的持续监控。

综上所述，面对日益严峻的微塑料污染问题及其对生态系统和人类健康的潜在威胁，未来检测技术的创新应重点聚焦高灵敏度色谱-质谱技术、光谱技术与纳米技术的深度融合，推进多技术联合应用，加快低成本检测设备的开发，构建智能化监测体系。这将为微塑料污染的有效防控提供坚实的科学依据，并为全球生态环境保护与公共健康保障提供强有力的技术支撑^［98］^。
